# Hemodynamic phenotypes defined by arterial stiffness index and pulse pressure with risk of diabetic microvascular complications in type 2 diabetes

**DOI:** 10.3389/fendo.2026.1917833

**Published:** 2026-08-11

**Authors:** Aochuan Sun, Hao Liu, Xusheng Yang, Hongling Guo, Ruowei Zeng, Yu Deng, Zhengtang Liu

**Affiliations:** 1 Department of Geriatrics, Xiyuan Hospital, China Academy of Chinese Medical Sciences, Beijing, China; 2 Department of Nephrology, Beijing Rehabilitation Hospital, Capital Medical University, Beijing, China; 3 Faculty of Chinese Medicine, Macau University of Science and Technology, Macao, Macao SAR, China; 4 Graduate School, Beijing University of Chinese Medicine, Beijing, China

**Keywords:** arterial stiffness index, diabetic microvascular complication, hemodynamic phenotype, pulse pressure, UK Biobank

## Abstract

**Objective:**

To identify hemodynamic phenotypes based on arterial stiffness index (ASI) and pulse pressure (PP) and examine their associations with incident diabetic microvascular complications (DMC) in individuals with type 2 diabetes (T2D).

**Methods:**

A total of 9,163 participants with T2D free of DMC at baseline were included from the UK Biobank. K-means clustering based on ASI and PP was used to identify hemodynamic phenotypes. Associations with incident DMC were evaluated using cause-specific Cox and Fine–Gray models. Restricted cubic spline analyses assessed nonlinear associations. Secondary analyses examined diabetic kidney disease (DKD), diabetic retinopathy (DR), and diabetic neuropathy (DN).

**Results:**

During a median follow-up of 12.9 years, 2,349 participants developed DMC. Three phenotypes were identified. Compared with the low ASI–low PP phenotype, the high PP phenotype was associated with a higher risk of DMC in both cause-specific Cox models (hazard ratio [HR] 1.16, 95% CI 1.05–1.28) and Fine–Gray models (subdistribution hazard ratio [SHR] 1.16, 95% CI 1.05–1.29), whereas no significant association was observed for the high ASI phenotype. ASI and PP showed L-shaped and J-shaped associations with DMC, respectively. ASI was primarily associated with DKD, whereas PP was associated with both DKD and DR.

**Conclusions:**

Hemodynamic phenotypes defined by ASI and PP were associated with differential risks of DMC in T2D. Elevated PP, rather than elevated ASI, was consistently associated with increased microvascular risk. Distinct associations of ASI and PP suggest heterogeneous hemodynamic pathways underlying DMC.

## 1 Introduction

Diabetic microvascular complications (DMC), including diabetic kidney disease (DKD), diabetic retinopathy (DR), and diabetic neuropathy (DN), remain major causes of morbidity and disability among individuals with type 2 diabetes (T2D) (1, 2). Despite substantial improvements in glycemic and cardiovascular risk factor management, considerable residual risk persists (3–5), suggesting that additional pathophysiological mechanisms contribute to disease development.

Hemodynamic alterations have emerged as potential contributors to microvascular injury (6). Under physiological conditions, elastic large arteries buffer pulsatile energy and protect the microcirculation from excessive hemodynamic stress. Arterial stiffening impairs this buffering function, increasing transmission of pulsatile pressure to vulnerable microvascular beds (7). Arterial stiffness index (ASI), a noninvasive surrogate of arterial stiffness, has shown good agreement with gold standard pulse wave velocity (PWV) (8, 9). Pulse pressure (PP), an indicator of pulsatile hemodynamic load, capture complementary aspects of vascular function and may jointly influence microvascular health (10).

Previous studies have reported associations between arterial stiffness and diabetic microvascular disease (11–14). However, most investigations have focused on individual vascular markers and assumed linear relationships. Such approaches may not adequately characterize the interplay between arterial stiffness and pulsatile load. Moreover, emerging evidence suggests that vascular risk factors may exhibit nonlinear associations with diabetes-related outcomes (15, 16), yet these relationships remain poorly understood in the context of DMC. Whether distinct hemodynamic phenotypes defined by ASI and PP confer differential risks of DMC is unknown.

Therefore, using data from the UK Biobank, we identified hemodynamic phenotypes based on ASI and PP through K-means clustering and examined their associations with incident DMC. We further evaluated the independent and nonlinear associations of ASI and PP with DMC to better characterize the hemodynamic mechanisms underlying diabetic microvascular complications.

## 2 Methods

### 2.1 Study design and population

This is a prospective cohort study based on the UK Biobank, a large-scale, population-based cohort that recruited over 500,000 middle-aged and older adults from 22 assessment centers across England, Scotland, and Wales between 2006 and 2010 (1). Ethical approval was obtained from the North West Multicenter Research Ethics Committee (reference number 11/NW/0382), and all participants provided written informed consent. The current study was conducted under UK Biobank application number 1222281, in full compliance with its data access policies and ethical regulations.

T2D at baseline was identified using the UK Biobank algorithm developed by Eastwood et al., which integrates self-reported diagnoses, hospital records, and medication history and has been validated with high reliability (17). We further incorporated glycated hemoglobin (HbA1c) from biochemical examinations, applying a threshold of 48 mmol/mol (6.5%) for diabetes diagnosis (18). After excluding participants who were lost to follow-up (n = 1296), 28,550 participants with T2D remained at baseline. From these, we further excluded those with any DMC at baseline (n = 1772) and those with missing data required for ASI and PP calculation (n = 17,615), leaving a total of 9163 participants for the final analysis (**Supplementary Figure 1**).

### 2.2 Assessment of arterial stiffness index and pulse pressure

ASI was assessed in 169,829 UK Biobank participants (2009–2010) using the PulseTrace PCA2 device with finger photoplethysmography. The peak-to-peak time (PPT) between the systolic and second diastolic peaks reflects pulse wave transit time to the lower body (19, 20). If the waveform was inadequate, the measurement was repeated on a larger finger or thumb. Standing height was measured with a Seca 202 stadiometer and manually entered. ASI was calculated as height (m) divided by PPT (s). This operator-independent method has been validated against carotid-femoral pulse wave velocity (9, 19, 21).

Systolic blood pressure (SBP) and diastolic blood pressure (DBP) were measured twice by trained nurses using an automated Omron 705 IT electronic blood pressure monitor after the participant had rested for at least 5 min in the seated position (15). PP was calculated as SBP minus DBP (mmHg) for each individual reading, and the resulting PP values were averaged for analysis.

### 2.3 Hemodynamic phenotype identification

Hemodynamic phenotypes were identified using K-means clustering based on baseline ASI and PP. Prior to clustering, both variables were standardized using z-score transformation to eliminate differences in scale. To determine the optimal number of clusters (K), three complementary approaches were applied, including the elbow method, silhouette analysis, and the gap statistic (22). The elbow method evaluated the within-cluster sum of squares across different values of K, whereas silhouette analysis assessed cluster cohesion and separation. The gap statistic compared the observed clustering performance with that expected under a reference null distribution. The optimal number of clusters was selected based on the overall agreement among these criteria (23). Based on these analyses, a three-cluster solution was chosen and used to define distinct hemodynamic phenotypes. Participants were subsequently assigned to clusters according to their nearest cluster centroid.

### 2.4 Ascertainment of outcomes

The primary outcome of this study was new-onset DMC, defined as a composite of the first occurrence of DKD, DN, or DR. Secondary outcomes were each DMC subtype individually. Events were identified by linkage to cumulative hospital inpatient records and the national death registry (24). Diagnoses were defined according to the International Classification of Diseases, Tenth Revision (ICD-10) and, where available, supplemented by self-reported data fields containing specific selection, disease, or procedure codes (**Supplementary Table 1**). Incident DMC was distinguished from baseline DMC by comparing the date of first diagnosis with the baseline assessment date; participants whose first DMC diagnosis occurred after baseline were classified as new-onset cases. Follow-up time was calculated from the baseline date until the first occurrence of a DMC event, death, or administrative censoring, whichever occurred first.

### 2.5 Assessment of covariates

Baseline covariates, collected via touchscreen questionnaire, covered sociodemographic factors, lifestyle, and medical history. Self-reported data included age, sex (female/male), ethnicity (White, Mixed, Asian/Asian British, Black/Black British, Chinese, or Other), and education (university/college degree, other qualifications, or none). The Townsend Deprivation Index was derived from postal codes; higher values indicate greater deprivation. Smoking status was categorized as never, former, or current; alcohol intake as ≥3 times/week, <3 times/week, or not current. BMI was weight (kg)/height² (m). Blood biomarkers comprised albumin (g/L), urea (mmol/L), cholesterol (mmol/L), creatinine (μmol/L), C-reactive protein (mg/L), and HbA1c (%). Comorbidity count was based on self-reported long-term conditions (**Supplementary Table 2**).

### 2.6 Statistical analysis

To address missing data, multiple imputation was performed using chained equations with five imputed datasets. Baseline characteristics were summarized according to hemodynamic phenotype groups. Continuous variables are presented as mean ± standard deviation (SD) or median (interquartile range [IQR]) as appropriate and were compared using one-way analysis of variance (ANOVA) or the Kruskal–Wallis test. Categorical variables are presented as frequencies (percentages) and were compared using the χ² test.

Incident DMC was the primary outcome. Analyses of individual DMC subtypes were conducted as secondary outcomes. All-cause death was treated as a competing event. Associations between hemodynamic phenotypes and incident DMC were evaluated using two complementary competing-risk approaches. First, cause-specific Cox proportional hazards models were fitted to estimate the instantaneous hazard of DMC, treating death as a censoring event. Results are presented as cause-specific hazard ratios (csHRs) with 95% confidence intervals (CIs). Second, Fine–Gray subdistribution hazard models were used to account for the competing risk of death, and results are reported as subdistribution hazard ratios (SHRs) with corresponding 95% CIs.

Associations of ASI and PP with DMC were examined using both continuous and categorical analyses. ASI was modeled per 1 m/s increase and by tertiles, whereas PP was modeled per 10-mmHg increase and by tertiles. To characterize dose–response relationships and assess potential nonlinearity, restricted cubic spline (RCS) analyses were performed within the cause-specific Cox framework. Three knots were placed at the 5th, 50th, and 95th percentiles of the exposure distributions. Nonlinearity was evaluated using likelihood ratio tests comparing models with and without spline terms. Cumulative incidence function (CIF) curves were generated to visualize the cumulative incidence of DMC and the competing event of death according to hemodynamic phenotype groups and tertiles of ASI and PP. Differences between groups were assessed using Gray’s test. To further investigate outcome-specific associations, separate analyses were conducted for DKD, DN, and DR using the same modeling strategy. Three hierarchical models were constructed. Model 1 was adjusted for age, sex, and ethnic groups; Model 2 was adjusted for drinking status, smoking status, educational level, Townsend Deprivation Index, and body mass index; Model 3 was further adjusted for albumin, urea, cholesterol, creatinine, C-reactive protein, HbA1c, and number of long-term conditions.

Several sensitivity analyses were conducted to evaluate the robustness of the primary findings. First, analyses were repeated after excluding participants with missing covariates to assess the impact of incomplete data. Second, analyses were repeated after excluding individuals who developed DMC within the first 2 years of follow-up to minimize potential reverse causation and protopathic bias. Third, subgroup analyses were performed to explore potential effect modification. Associations between hemodynamic phenotypes and risk of DMC were stratified by sex (male vs. female), age (<65 vs. ≥65 years), HbA1c (<48 vs. ≥48 mmol/mol), history of hypertension (yes vs. no), and history of coronary heart disease (yes vs. no). Interaction terms between exposures and subgroup variables were introduced into multivariable Cox proportional hazards models, and *P* for interaction was calculated. Finally, we compared the baseline characteristics between participants who were excluded from the analysis due to missing ASI or PP measurements and those who were included in the analysis.

The proportional hazards assumption was evaluated using Schoenfeld residuals. All statistical analyses were performed using R software (version 4.4.1; R Foundation for Statistical Computing, Vienna, Austria). All tests were two-sided, and a *P* value < 0.05 was considered statistically significant.

## 3 Results

### 3.1 Participant characteristics

Baseline characteristics according to hemodynamic phenotype groups are presented in **Table 1**. After excluding participants with prevalent DMC at baseline, a total of 9,163 individuals with T2D were included in the analysis. The mean age was 59.7 ± 7.1 years, and 38.3% were women. During a median follow-up of 12.9 years, 2,349 participants developed incident DMC. Compared with participants in Cluster 1, those in Cluster 2 and Cluster 3 were generally older, reported more frequent alcohol consumption, had lower educational attainment, were likely to be more affluent. Significant differences were observed across clusters for most clinical and biochemical characteristics.

**Table 1:** Baseline characteristics of the study population according to hemodynamic phenotype groups.

Participant characteristics	Total	Hemodynamic phenotype groups			*P* value
		Cluster 1 (Low ASI-PP)	Cluster 2 (High ASI)	Cluster 3 (High PP)	
No. of participants	9163	4400	2260	2503	
Age, y	59.7 ± 7.1	57.3 ± 7.4	61.4 ± 6.2	62.3 ± 5.7	< 0.001
Gender, n (%)					< 0.001
Female	3506 (38.3)	1791 (40.7)	684 (30.3)	1031 (41.2)	
Male	5657 (61.7)	2609 (59.3)	1576 (69.7)	1472 (58.8)	
Ethnic groups, n (%)					< 0.001
White	7246 (79.1)	3307 (75.2)	1873 (82.9)	2066 (82.5)	
Mix	82 (0.9)	45 (1)	18 (0.8)	19 (0.8)	
Asian or Asian British	1022 (11.2)	597 (13.6)	193 (8.5)	232 (9.3)	
Black or Black British	525 (5.7)	292 (6.6)	104 (4.6)	129 (5.2)	
Chinese	37 (0.4)	19 (0.4)	14 (0.6)	4 (0.2)	
Others	251 (2.7)	140 (3.2)	58 (2.6)	53 (2.1)	
Drinking status, n (%)					< 0.001
≥ 3 times per week	2716 (29.6)	1148 (26.1)	754 (33.4)	814 (32.5)	
< 3 times per week	3141 (34.3)	1547 (35.2)	769 (34)	825 (33)	
Not current	3306 (36.1)	1705 (38.8)	737 (32.6)	864 (34.5)	
Smoking status, n (%)					< 0.001
Non-smoker	4441 (48.5)	2288 (52)	915 (40.5)	1238 (49.5)	
Former smoker	3740 (40.8)	1605 (36.5)	1065 (47.1)	1070 (42.7)	
Current smoker	982 (10.7)	507 (11.5)	280 (12.4)	195 (7.8)	
Educational level, n (%)					< 0.001
College or University degree	2469 (26.9)	1291 (29.3)	540 (23.9)	638 (25.5)	
Any other qualification	4441 (48.5)	2177 (49.5)	1074 (47.5)	1190 (47.5)	
No qualification	2253 (24.6)	932 (21.2)	646 (28.6)	675 (27)	
Townsend deprivation index	-0.8 (-2.9, 2.2)	-0.5 (-2.7, 2.5)	-1.1 (-3.0, 2.0)	-1.0 (-3.0, 1.7)	< 0.001
BMI, (kg/m^2^)	31.3 ± 5.6	31.7 ± 5.9	30.7 ± 5.0	31.0 ± 5.6	< 0.001
Albumin, (g/L)	45.2 ± 2.8	45.0 ± 2.8	45.3 ± 2.7	45.2 ± 2.8	< 0.001
Urea, (mmol/L)	5.6 ± 1.6	5.5 ± 1.6	5.6 ± 1.5	5.8 ± 1.8	< 0.001
Cholesterol, (mmol/L)	4.7 ± 1.1	4.7 ± 1.1	4.7 ± 1.1	4.6 ± 1.1	0.855
Creatinine, (umol/L)	73.9 ± 19.1	73.0 ± 18.0	74.6 ± 17.0	74.6 ± 22.6	< 0.001
C-reactive protein, (mg/L)	2.0 (0.9, 4.1)	2.1 (1.0, 4.4)	1.8 (0.9, 3.8)	1.9 (0.9, 3.8)	< 0.001
HbA1c, (mmol/mol)	53.0 ± 13.9	53.6 ± 14.6	53.0 ± 13.3	52.1 ± 13.2	< 0.001
No. of long-term conditions, n (%)					< 0.001
0	1491 (16.3)	836 (19)	341 (15.1)	314 (12.5)	
1-3	5539 (60.4)	2556 (58.1)	1403 (62.1)	1580 (63.1)	
≥ 3	2133 (23.3)	1008 (22.9)	516 (22.8)	609 (24.3)	
ASI, (m/s)	10.0 ± 3.1	8.8 ± 2.0	14.0 ± 2.4	8.4 ± 2.2	< 0.001
PP, (mmHg)	61.9 ± 15.7	50.8 ± 8.3	64.4 ± 12.5	78.9 ± 11.7	< 0.001
Diabetic microvascular complications, n (%)	2349 (25.6)	1014 (23)	562 (24.9)	773 (30.9)	< 0.001
Diabetic kidney disease, n (%)	1319 (14.4)	538 (12.2)	317 (14)	464 (18.5)	< 0.001
Diabetic neuropathy, n (%)	446 (4.9)	220 (5)	114 (5)	112 (4.5)	0.562
Diabetic retinopathy, n (%)	1157 (12.6)	490 (11.1)	280 (12.4)	387 (15.5)	< 0.001

Continuous variables are presented as mean ± standard deviation and median (interquartile range), and categorical variables as counts (percentages). BMI, body mass index; HbA1c, glycated hemoglobin A1c; ASI, arterial stiffness index; PP, pulse pressure. Data after multiple imputation.

### 3.2 Identification of hemodynamic phenotypes

**Figure 1:**
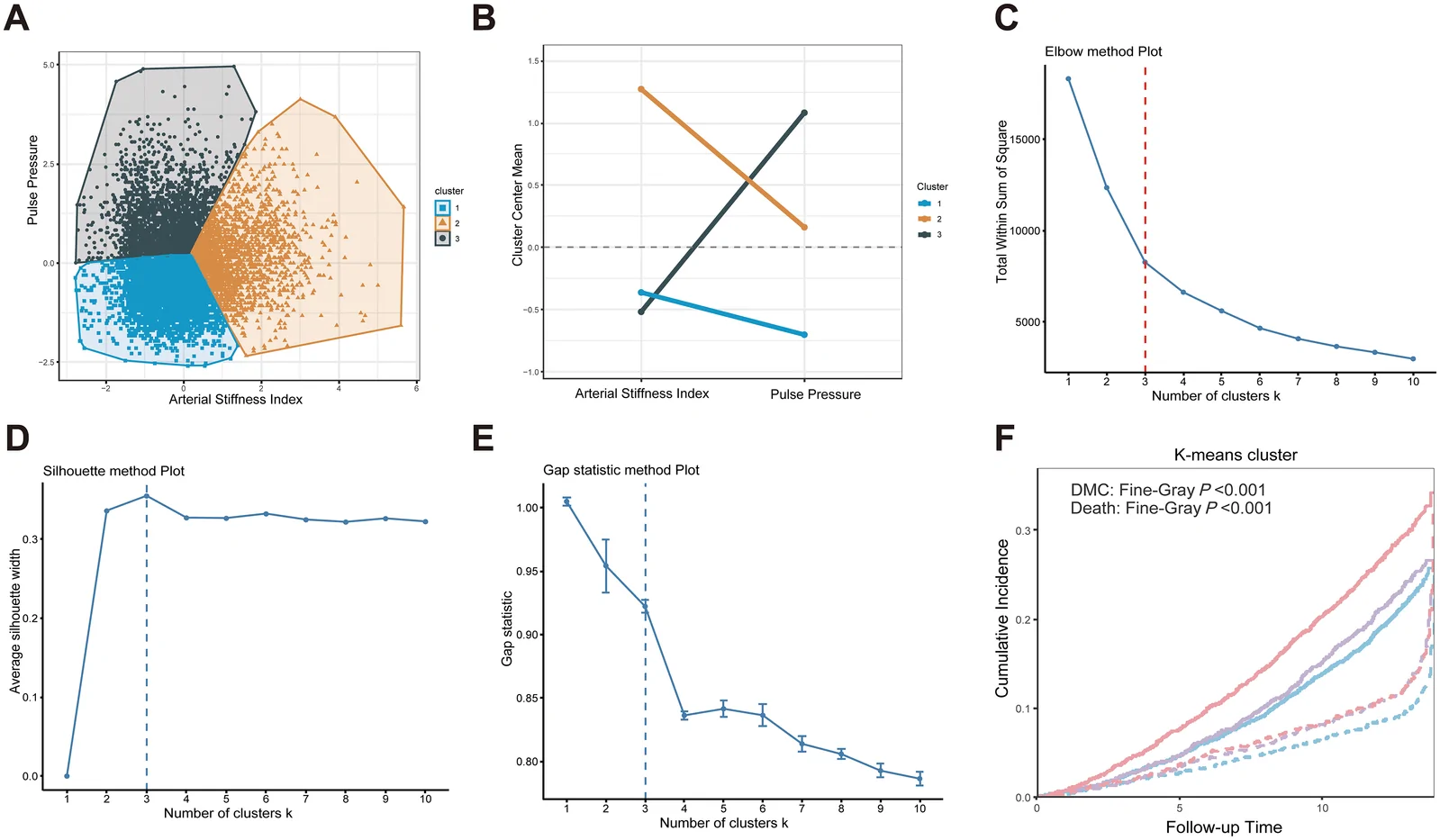
Identification of hemodynamic phenotypes based on arterial stiffness index and pulse pressure and their association with diabetic microvascular complications. **(A)** Convex hull plot and **(B)** parallel coordinates plot illustrating three hemodynamic phenotypes identified by K-means clustering based on standardized arterial stiffness index (ASI) and pulse pressure (PP). The optimal number of clusters was determined using the **(C)** elbow method, **(D)** silhouette analysis, and **(E)** gap statistic, all of which supported a three-cluster solution. **(F)** Cumulative incidence function (CIF) curves for diabetic microvascular complications and competing risk of death according to cluster membership. Solid and dashed lines represent DMC and death, respectively. Colors indicate clusters: blue (Cluster 1, low), purple (Cluster 2, middle), and pink (Cluster 3, high). Gray’s test P values are shown.

K-means clustering based on standardized ASI and pulse pressure identified three distinct hemodynamic phenotypes (**Figures 1A, B**). The optimal number of clusters was determined using the elbow method, silhouette analysis, and the gap statistic. The elbow method revealed a distinct inflection at k = 3 (**Figure 1C**). Silhouette analysis yielded a maximum mean silhouette width of 0.35 at k = 3 (compared to 0.33 for k = 2 and 0.32 for k = 4) (**Figure 1D**). Following the criterion Gap(k) ≥ Gap(k+1) - SE(k+1), k = 3 was the first cluster number satisfying this condition (**Figure 1E**; **Supplementary Table 3**). All three methods consistently converged on a three-cluster solution, which was adopted for subsequent analyses. Accordingly, three phenotypes were defined as: low ASI–low PP (Cluster 1), high ASI (Cluster 2), and high PP (Cluster 3).

### 3.3 Associations between hemodynamic phenotypes and risk of DMC

CIF curves showed significant differences in the cumulative incidence of DMC across the three hemodynamic phenotypes (Gray’s test *P* < 0.001). Significant differences were also observed for the competing event of death (Gray’s test *P* < 0.001) (**Figure 1F**).

In cause-specific Cox models, participants in the high PP phenotype exhibited a significantly higher risk of incident DMC compared with those in the low ASI–PP phenotype. In the fully adjusted model (Model 3), the adjusted HR was 1.16 (95% CI 1.05–1.28). By contrast, the association between the high ASI phenotype and DMC was attenuated after adjustment for potential confounders and was no longer statistically significant (HR 0.96, 95% CI 0.86–1.07) (**Table 2**).

**Table 2:** Associations of hemodynamic phenotypes with diabetic microvascular complications in cause-specific and competing risk models.

Hemodynamic phenotypes	Adjusted hazard ratio (95% CI)		
	Model 1	Model 2	Model 3
Cause-specific hazard model (Events/Total)			
Low ASI-PP (1014/4400)	1 (Ref)	1 (Ref)	1 (Ref)
High ASI (562/2260)	0.89 (0.8, 0.99)	0.90 (0.81, 0.99)	0.96 (0.86, 1.07)
High PP (773/2503)	1.13 (1.03, 1.25)*	1.16 (1.05, 1.28)*	1.16 (1.05, 1.28)*
Fine-Gray subdistribution model (Deaths/Total)			
Low ASI-PP (1373/4400)	1 (Ref)	1 (Ref)	1 (Ref)
High ASI (773/2260)	0.9 (0.81, 0.99)	0.91 (0.82, 1.01)	0.99 (0.89, 1.1)
High PP (357/2503)	1.12 (1.02, 1.24)*	1.15 (1.04, 1.27)*	1.16 (1.05, 1.29)*

Model 1 was adjusted for age, sex, and ethnic groups; Model 2 was adjusted for drinking status, smoking status, educational level, Townsend Deprivation Index, and body mass index; Model 3 was further adjusted for albumin, urea, cholesterol, creatinine, C-reactive protein, glycated hemoglobin, and number of long-term conditions. Ref, reference; ASI, arterial stiffness index; PP, pulse pressure. Cause-specific hazard ratios were estimated using Cox proportional hazards models. Subdistribution hazard ratios were estimated using Fine–Gray models accounting for the competing risk of death.* P < 0.05.

Similar findings were observed in competing-risk analyses accounting for death as a competing event. In the fully adjusted Fine–Gray model, the high PP phenotype remained associated with a higher subdistribution hazard of DMC (SHR 1.16, 95% CI 1.05–1.29), whereas no significant association was observed for the high ASI phenotype (SHR 0.99, 95% CI 0.89–1.10) (**Table 2**).

### 3.4 Associations of ASI and PP with DMC

**Figure 2:**
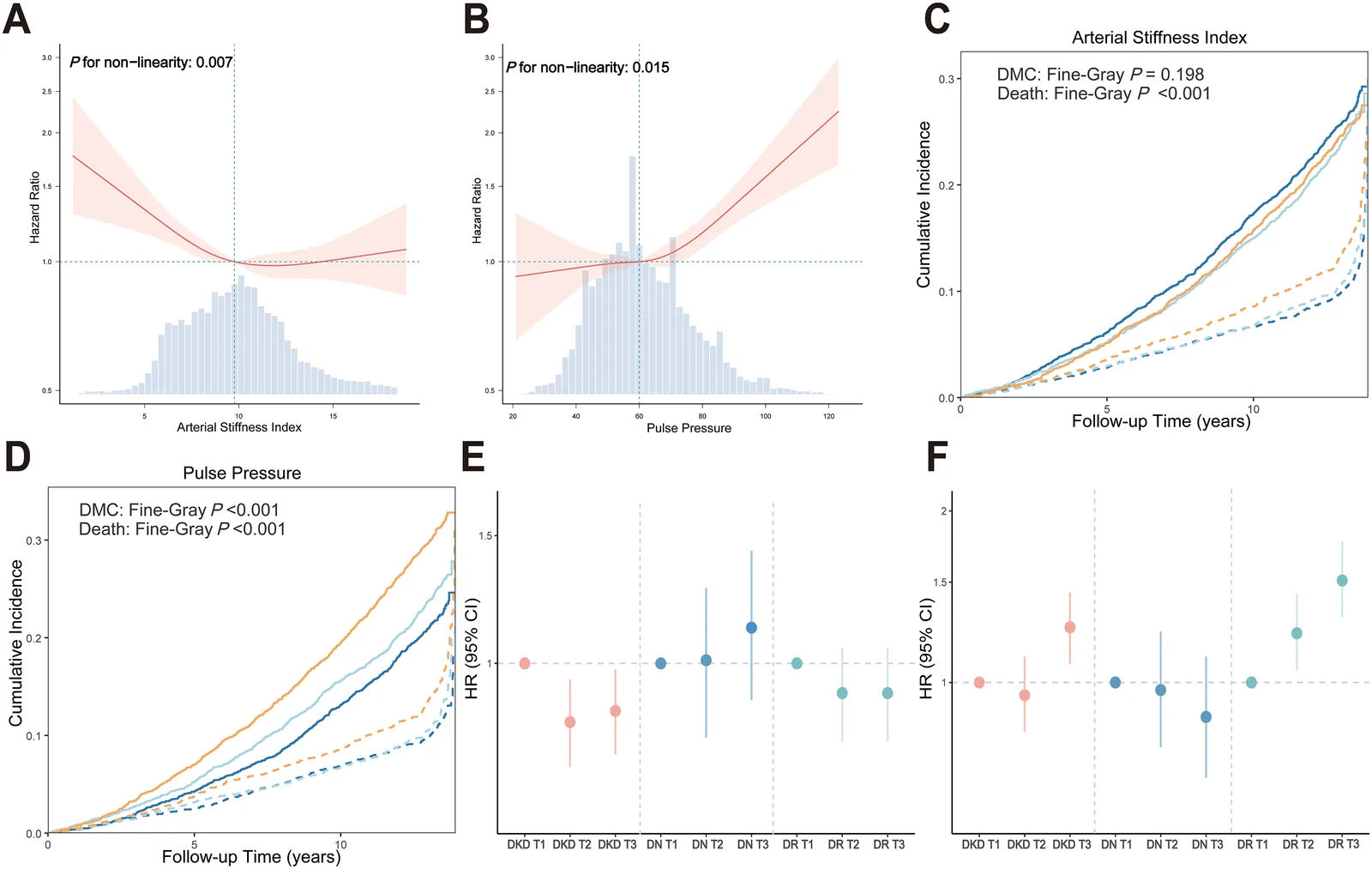
Associations of arterial stiffness index and pulse pressure with diabetic microvascular complications and their subtypes. **(A)** Restricted cubic spline (RCS) analysis for arterial stiffness index (ASI) and incident diabetic microvascular complications (DMC). **(B)** RCS analysis for pulse pressure (PP) and incident DMC. Solid lines indicate adjusted hazard ratios (HRs), and shaded areas represent 95% confidence intervals (CIs). **(C)** Cumulative incidence function (CIF) curves for DMC and competing risk of death according to ASI tertiles. **(D)** CIF curves for DMC and competing risk of death according to PP tertiles. Solid and dashed lines represent DMC and death, respectively. Colors denote tertiles: blue (Tertile 1, low), light blue (Tertile 2, middle), and orange (Tertile 3, high). Gray’s test P values are shown. **(E)** Adjusted HRs for diabetic kidney disease (DKD), diabetic neuropathy (DN), and diabetic retinopathy (DR) across ASI tertiles. **(F)** Adjusted HRs for DKD, DN, and DR across PP tertiles. All adjusted analyses were performed using multivariable Model 3 Cox proportional hazards models.

Restricted cubic spline analyses revealed significant nonlinear associations between both ASI and PP with incident DMC (**Figures 2A, B**). For ASI, an L-shaped relationship was observed (*P* for nonlinearity = 0.007). DMC risk decreased with increasing ASI up to the inflection point of 9.92 m/s (HR 0.945, 95% CI 0.912–0.978), beyond which the risk plateaued (HR 1.015, 95% CI 0.988–1.043) (**Supplementary Table 4**). For PP, a J-shaped association was found (*P* for nonlinearity = 0.015). Risk remained relatively stable below 71.2 mmHg (HR per 10 mmHg 1.036, 95% CI 0.983–1.091) but increased significantly above this threshold (HR 1.161, 95% CI 1.072–1.258) (**Supplementary Table 5**).

Consistent patterns were observed in cumulative incidence analyses across ASI and PP tertiles (**Figures 2C, D**). Significant differences in the cumulative incidence of DMC were observed across PP tertiles (Gray’s test *P* < 0.001), whereas weaker differences were observed across ASI tertiles (Gray’s test *P* = 0.198).

In the fully adjusted Cox model (Model 3), each 1 m/s increase in ASI was associated with 2% lower risk of DMC (HR 0.98, 95% CI 0.97–0.99). Each 10-mmHg increase in pulse pressure was associated with 8% higher risk of DMC (HR 1.08, 95% CI 1.05–1.10). Compared with participants in the lowest tertile, those in the highest tertile of PP had a significantly increased risk of DMC (HR 1.23, 95% CI 1.11–1.37). For ASI, the association across tertiles was attenuated after multivariable adjustment but remained significant. Compared with the lowest tertile, the HRs for the second and third tertiles were both 0.87 (**Table 3**). In competing risk analyses accounting for death, the results remained consistent with the primary findings (**Supplementary Table 6**).

**Table 3:** Associations of arterial stiffness index and pulse pressure with diabetic microvascular complications.

Variable (events/total)	Model 1		Model 2		Model 3	
	HR (95% CI)	*P* value	HR (95% CI)	*P* value	HR (95% CI)	*P* value
Arterial stiffness index						
Increase per 1 m/s	0.98 (0.96, 0.99)	0.001	0.97 (0.96, 0.99)	<0.001	0.98 (0.97, 0.99)	0.013
Tertile 1 (818/3052)	1 (Ref)		1 (Ref)		1 (Ref)	
Tertile 2 (759/3053)	0.86 (0.78, 0.95)	0.004	0.83 (0.75, 0.92)	<0.001	0.87 (0.79, 0.97)	0.009
Tertile 3 (772/3058)	0.82 (0.74, 0.91)	<0.001	0.80 (0.72, 0.89)	<0.001	0.87 (0.79, 0.97)	0.01
*P* for trend		<0.001		<0.001		0.011
Pulse pressure						
Increase per 10 mmHg	1.06 (1.03, 1.08)	<0.001	1.06 (1.03, 1.09)	<0.001	1.08 (1.05, 1.10)	<0.001
Tertile 1 (642/2967)	1 (Ref)		1 (Ref)		1 (Ref)	
Tertile 2 (743/3039)	0.98 (0.88, 1.09)	0.748	1.01 (0.9, 1.12)	0.916	1.05 (0.94, 1.17)	0.389
Tertile 3 (964/3157)	1.13 (1.02, 1.26)	0.024	1.16 (1.05, 1.29)	0.005	1.23 (1.11, 1.37)	<0.001
*P* for trend		0.015		0.003		<0.001

Model 1 was adjusted for age, sex, and ethnic groups; Model 2 was adjusted for drinking status, smoking status, educational level, Townsend Deprivation Index, and body mass index; Model 3 was further adjusted for albumin, urea, cholesterol, creatinine, C-reactive protein, glycated hemoglobin, and number of long-term conditions. HR, hazard ratio; CI, confidence interval; Ref, reference. Hazard ratios were estimated using multivariable Cox proportional hazards models.

### 3.5 Associations with individual microvascular outcomes

Associations between ASI, PP, and individual microvascular outcomes are shown in **Figures 2E, F**. For ASI, significant associations were primarily observed for DKD. A nonlinear L-shaped relationship was identified between ASI and DKD (**Supplementary Figure 2**), with an inflection point at approximately 11.53 m/s (**Supplementary Table 7**). No significant associations were observed between ASI and DN or DR (**Supplementary Figures 3**, **S4**). For PP, distinct association patterns were observed across microvascular outcomes. PP showed a J-shaped association with DKD (**Supplementary Figure 5**), with an inflection point at approximately 57 mmHg (**Supplementary Table 8**), and a positive linear association with DR (**Supplementary Figure 6**). In contrast, no significant association was observed between PP and DN (**Supplementary Figure 7**).

### 3.6 Sensitivity analyses

Results from sensitivity analyses were generally consistent with the primary findings. First, in the complete-case analysis excluding participants with missing covariates, the associations between hemodynamic phenotypes, ASI, PP, and DMC risk remained similar (**Supplementary Tables 9**, **S10**). Second, exclusion of participants who developed DMC within the first 2 years of follow-up did not materially alter the observed associations (**Supplementary Tables 11**, **S12**). Third, subgroup analyses stratified by sex, age, HbA1c level, hypertension status, and coronary heart disease status demonstrated broadly consistent associations across subgroups. No significant interactions were observed (all *P* for interaction > 0.05) (**Supplementary Table 13**). Baseline characteristics of participants included in the analysis and those excluded because of unavailable ASI or PP measurements are presented in **Supplementary Table 14**. Overall, the two groups were broadly comparable with respect to age and sex, although modest differences were observed in several demographic and clinical characteristics.

## 4 Discussion

In this prospective cohort study of 9,163 individuals with T2D from the UK Biobank, we investigated the associations of hemodynamic phenotypes defined by ASI and PP with incident DMC. Three distinct hemodynamic phenotypes were identified, among which the high PP phenotype was consistently associated with an increased risk of DMC, whereas no independent association was observed for the high ASI phenotype after multivariable adjustment. When ASI and PP were examined separately, both exhibited significant nonlinear associations with DMC, characterized by an L-shaped relationship for ASI and a J-shaped relationship for PP. Analyses of individual microvascular outcomes further revealed that the association of ASI was primarily driven by DKD, whereas PP showed distinct associations with DKD and DR but not DN. Collectively, these findings suggest that hemodynamic phenotypes provide a clinically meaningful framework for risk stratification of DMC and indicate that pulsatile pressure load may be more strongly associated with microvascular risk than arterial stiffness alone in individuals with T2D.

The principal finding beyond the individual associations of ASI and PP is that integrating these two complementary hemodynamic markers identified clinically distinct phenotypes associated with differential risks of DMC. Although previous studies have demonstrated associations between arterial stiffness or pulse pressure and diabetic microvascular disease (25, 26), most evaluated these markers separately (13). By combining ASI and PP, our study captured different dimensions of arterial hemodynamics, including structural vascular properties and pulsatile pressure load, thereby providing a more comprehensive characterization of vascular function. Notably, only the high PP phenotype remained independently associated with DMC after multivariable adjustment, whereas the association for the high ASI phenotype was attenuated. These findings suggest that pulsatile hemodynamic load, rather than arterial stiffness alone, may play a more prominent role in the development of diabetic microvascular injury.

When ASI was analyzed as a continuous variable in Cox regression models, an inverse association with DMC risk was observed. However, this finding should not be interpreted as indicating a protective effect of higher ASI. Instead, the restricted cubic spline analysis revealed an L-shaped association, with risk decreasing across the lower ASI range and reaching a plateau at approximately 11.5 m/s. Thus, the inverse estimates obtained from linear modeling likely reflect the averaging of a nonlinear exposure–response relationship rather than a true inverse association. The biological basis of this nonlinear pattern remains to be fully elucidated. Although ASI has shown good agreement with carotid–femoral pulse wave velocity (cfPWV), it is derived from digital pulse contour analysis and may also capture aspects of peripheral vascular function and pulse wave transmission characteristics (27, 28). Therefore, ASI should be interpreted as a composite hemodynamic index rather than a direct equivalent of reference-standard arterial stiffness measurement. In addition, the plateau observed beyond the ASI threshold may partly reflect characteristics of the UK Biobank population. As UK Biobank participants are relatively young and generally healthier than the broader population, individuals with advanced arterial stiffening may be underrepresented, potentially limiting the observable risk gradient at higher ASI levels. Future studies using direct arterial stiffness measurements in more diverse populations are needed to determine whether this nonlinear association reflects underlying vascular physiology or characteristics of ASI measurement. Conversely, PP serves as a direct metric of pulsatile hemodynamics, offering a more sensitive reflection of the transmission efficiency of pressure waves from ventricular ejection to the distal microcirculation (29). As the terminal target of pressure wave propagation, the microvascular bed sustains damage that likely depends more on its actual exposure to pulsatile stress rather than the intrinsic stiffness of proximal large arteries (7, 30). This interpretation is further supported by the phenotype analyses, in which the high PP phenotype remained independently associated with DMC, whereas no independent association was observed for the high ASI phenotype after multivariable adjustment. Together, these findings indicate that pulsatile hemodynamic load may be a more proximal determinant of diabetic microvascular injury than arterial stiffness alone in individuals with T2D.

We further observed substantial heterogeneity across individual microvascular outcomes. The association between ASI and DMC was primarily driven by DKD, whereas PP was associated with both DKD and DR but not DN. For DKD, our results parallel previous evidence (31–33). As a high-flow, low-resistance organ, the kidney’s glomerular capillaries face direct exposure to systemic pressure, relying heavily on upstream buffering (34). Elevated arterial stiffness facilitates pulsatile wave transmission into glomeruli (35), subjecting nephrons to chronic mechanical stress (36). Impaired renal autoregulation amplifies this pulsatile energy, triggering hyperfiltration, glomerulosclerosis, and functional decline. In chronic kidney disease, premature vascular aging and calcification abolish or invert the stiffness gradient, dismantling microcirculatory protection (37). Consequently, pulsatile waves penetrate renal microvasculature deeply; upon afferent arteriole failure, glomeruli endure direct damage (38). For DR, PP showed a positive linear association with DR risk (39, 40), while previous studies have also reported U-shaped or J-shaped relationships (41). Retinal microvessels, as terminal vascular units, lack robust upstream buffering mechanisms (42). Retinopathy impairs vascular reactivity, where compromised autoregulation and pressure-dependent hyperperfusion accelerate lesion progression (43). This diminished autoregulatory capacity heightens retinal vulnerability to hemodynamic fluctuations (44). Conversely, neither ASI nor PP exhibited significant associations with DN, mirroring inconsistencies in existing literature: while arterial stiffness increases in DN, its independent pathogenicity and temporal relationships remain undetermined (45). This null finding may reflect stronger autoregulation of the vasa nervorum (46), the predominant influence of metabolic disturbance, chronic ischemia, and inflammation (47), and the relatively limited hemodynamic contribution (48). Additionally, limited DN events in our cohort might constrain statistical power. Ultimately, hemodynamic perturbations exert heterogeneous effects across microvascular targets. Characterized by high perfusion, low resistance, and profound reliance on proximal buffering, the kidney exhibits paramount sensitivity to arterial stiffness and pulsatile transmission.

This study has several strengths, including its prospective design, large sample size, and integration of ASI and PP into clinically interpretable hemodynamic phenotypes. Several limitations should also be acknowledged. First, although individuals with prior DMC and those diagnosed within a 2-year washout period were excluded, the possibility of reverse causality cannot be entirely ruled out. Second, although we adjusted for a broad range of established risk factors for DMC, residual confounding cannot be completely excluded. In particular, diabetes duration and longitudinal medication exposure, including antihypertensive, glucose-lowering, and lipid-lowering therapies, were not incorporated into the analyses. Further studies with more detailed information on disease duration and treatment history are warranted to clarify their potential influence on the observed associations. Third, although ASI has been demonstrated to correlate highly with PWV, it is not the gold standard. Fourth, both ASI and PP were measured only once at baseline, precluding assessment of the dynamic effects of their longitudinal changes. Fifth, the relatively small number of diabetic neuropathy events may have limited statistical power and contributed to the null findings. Sixth, the limited ethnic diversity of the UK Biobank population may restrict the generalizability of these conclusions. Finally, selection bias cannot be completely excluded because participants without ASI or PP measurements were excluded from the analyses. Although included and excluded participants were broadly comparable in terms of age and sex, modest differences in several baseline characteristics were observed. Therefore, the analytic cohort may not fully represent all individuals with T2D in the UK Biobank, which may limit the generalizability of our findings.

## 5 Conclusion

In conclusion, hemodynamic phenotypes defined by ASI and PP were associated with differential risks of diabetic microvascular complications in T2D. Elevated PP, rather than elevated ASI alone, was consistently associated with a higher risk of DMC. The distinct nonlinear associations observed for ASI and PP further suggest heterogeneous hemodynamic pathways underlying microvascular injury. These findings provide a novel framework for vascular risk stratification and may help identify individuals at increased risk of diabetic microvascular complications.

## Data Availability

Publicly available datasets were analyzed in this study. This data can be found here: Data from the UK Biobank are available to researchers upon application at https://www.ukbiobank.ac.uk. The analytic code and supporting materials used in this study will be made available by the corresponding author upon reasonable request.
