# Supplementary material for: Hemodynamic phenotypes defined by arterial stiffness index and pulse pressure with risk of diabetic microvascular complications in type 2 diabetes
Source: Front Endocrinol (Lausanne). 2026 Aug 11;17:1917833. doi: 10.3389/fendo.2026.1917833 (PMC13503228; doi:10.3389/fendo.2026.1917833)
Supplement: Supplementary file 1 [file DataSheet1.docx]

**Figure S1**. Research Flowchart

**Figure S2**. A nonlinear L-shaped relationship was identified between ASI and DKD

ASI, arterial stiffness index; DKD, diabetic kidney disease.

**Figure S3**. No significant associations were observed between ASI and DN

ASI, arterial stiffness index; DN, diabetic neuropathy.

**Figure S4**. No significant associations were observed between ASI and DR

ASI, arterial stiffness index; DR, diabetic retinopathy.

**Figure S5**. A nonlinear J-shaped relationship was identified between PP and DKD

PP, pulse pressure; DKD, diabetic kidney disease.

**Figure S6**. A positive linear relationship was identified between PP and DR

PP, pulse pressure; DR, diabetic retinopathy.

**Figure S7**. No significant associations were observed between PP and DN

PP, pulse pressure; DN, diabetic neuropathy.

**Table S1.** Definition of diabetic microvascular complications

|  | **ICD-10** | **Self-reported field IDs** |
| --- | --- | --- |
| Nephropathy | E112,E122,E132,E142, N083,N180,N181,N182, N183,N184,N185,N186, N187,N188,N189 | 20002(1607) |
| Retinopathy | E113,E123,E133,E143, H280,H360 | 20002(1276) |
| Neuropathy | E114,E124,E134,E144, G590,G629,G632,G990 | 20002(1468) |

ICD, International Classification of Diseases

**Table S2.** Definition and list of long-term morbidities.

|  | Morbidity grouping* | Conditions included | Code |
| --- | --- | --- | --- |
| 1 | Hypertension | Hypertension | 1065 |
|  |  | Essential hypertension | 1072 |
| 2 | Coronary heart disease | Heart attack/MI | 1075 |
|  |  | Angina | 1074 |
| 3 | Diabetes | Diabetic nephropathy | 1607 |
|  |  | Diabetic neuropathy/ulcers | 1468 |
|  |  | Diabetes | 1220 |
|  |  | Type 1 diabetes | 1222 |
|  |  | Type 2 diabetes | 1223 |
|  |  | Diabetic eye disease | 1276 |
| 4 | Stroke/TIA | Stroke | 1081 |
|  |  | TIA | 1082 |
|  |  | Subarachnoid haemorrhage | 1086 |
|  |  | Brain haemorrhage | 1491 |
|  |  | Ischaemic stroke | 1583 |
| 5 | Atrial fibrillation | Atrial fibrillation | 1471 |
| 6 | Heart failure | Cardiomyopathy | 1079 |
|  |  | Hypertrophic cardiomyopathy | 1588 |
|  |  | Heart failure/pulmonary oedema | 1076 |
| 7 | Peripheral vascular disease | Peripheral vascular disease | 1067 |
|  |  | Leg claudication/intermittent  claudication | 1087 |
| 8 | COPD | COPD/Chronic obstructive pulmonary  disease | 1112 |
|  |  | Emphysema/Chronic bronchitis | 1113 |
|  |  | Emphysema | 1472 |
| 9 | Asthma | Asthma | 1111 |
| 10 | Bronchiectasis | Bronchiectasis | 1114 |
| 11 | Cancer* | “yes”/”no” to “have you ever had  cancer?” |  |
| 12 | Dyspepsia | Gastro-oesophageal reflux (GORD) | 1138 |
|  |  | Oesophagitis/Barrett’s oesophagus | 1139 |
|  |  | Gastric stomach ulcers | 1142 |
|  |  | Gastric erosions/gastritis | 1143 |
|  |  | Duodenal ulcer | 1457 |
|  |  | Dyspepsia/indigestion | 1510 |
|  |  | Hiatus hernia | 1474 |
|  |  | Helicobacter pylori | 1442 |
| 13 | Diverticular disease | Diverticular disease/diverticulitis | 1458 |
| 14 | Irritable bowel syndrome | Irritable bowel syndrome | 1154 |
| 15 | Chronic liver disease | Oesophageal varices | 1141 |
|  |  | Non infective hepatitis | 1157 |
|  |  | Liver failure/cirrhosis | 1158 |
|  |  | Primary biliary cirrhosis | 1506 |
| 16 | Inflammatory bowel disease | Inflammatory bowel disease | 1461 |
|  |  | Crohn’s disease | 1462 |
|  |  | Ulcerative colitis | 1463 |
| 17 | Constipation | Constipation | 1599 |
| 18 | Viral hepatitis | Hepatitis B | 1579 |
|  |  | Hepatitis C | 1580 |
|  |  | Hepatitis D | 1581 |
| 19 | Depression | Depression | 1286 |
|  |  | Postnatal depression | 1531 |
| 20 | Anxiety | Anxiety/panic attacks | 1287 |
|  |  | Nervous breakdown | 1288 |
|  |  | Post-traumatic stress disorder | 1469 |
|  |  | Obsessive compulsive disorder | 1615 |
|  |  | Stress | 1614 |
|  |  | Insomnia | 1616 |
|  |  | Psychological/psychiatric problem | 1243 |
| 21 | Schizophrenia/Bipolar affective disorder | Schizophrenia | 1289 |
|  |  | Mania | 1291 |
|  |  | Bipolar disorder | 1291 |
|  |  | Manic depression | 1291 |
| 22 | Connective tissue diseases | Myositis/myopathy | 1322 |
|  |  | Systemic lupus erythematosus/SLE | 1381 |
|  |  | Connective tissue disorder | 1373 |
|  |  | Sjogren’s syndrome; Sicca syndrome | 1382 |
|  |  | Dermatopolymyositis | 1383 |
|  |  | Scleroderma/systemic sclerosis | 1384 |
|  |  | Rheumatoid arthritis | 1464 |
|  |  | Psoriatic arthropathy | 1477 |
|  |  | Dermatomyositis | 1480 |
|  |  | Polymyositis | 1481 |
|  |  | Polymyalgia rheumatica | 1377 |
| 23 | Painful conditions | Back pain | 1534 |
|  |  | Joint pain | 1537 |
|  |  | Headaches (not migraine) | 1436 |
|  |  | Sciatica | 1476 |
|  |  | Plantar fasciitis | 1540 |
|  |  | Carpal tunnel syndrome | 1541 |
|  |  | Fibromyalgia | 1542 |
|  |  | Arthritis | 1538 |
|  |  | Shingles | 1573 |
|  |  | Disc problem | 1532 |
|  |  | Prolapsed disc/slipped disc | 1312 |
|  |  | Spine arthritis/spondylitis | 1311 |
|  |  | Ankylosing spondylitis | 1313 |
|  |  | Back problem | 1294 |
|  |  | Osteoarthritis | 1465 |
|  |  | Gout | 1466 |
|  |  | Cervical spondylosis | 1478 |
|  |  | Trigeminal neuralgia | 1523 |
|  |  | Disc degeneration | 1533 |
|  |  | Trapped nerve/compressed nerve | 1257 |
| 24 | Osteoporosis | Osteoporosis | 1309 |
| 25 | Thyroid disorders | Thyroid problem (not cancer） | 1224 |
|  |  | Hyperthyroidism/thyrotoxicosis | 1225 |
|  |  | Hypothyroidism/myxoedema | 1226 |
|  |  | Graves disease | 1522 |
|  |  | Thyroid goitre | 1610 |
|  |  | Thyroiditis | 1428 |
| 26 | Alcohol problems | Alcohol dependency | 1408 |
|  |  | Alcoholic liver disease/alcoholic  cirrhosis | 1604 |
| 27 | Chronic kidney disease | Polycystic kidney | 1427 |
|  |  | Diabetic nephropathy | 1607 |
|  |  | Renal/kidney failure | 1192 |
|  |  | Renal failure requiring dialysis | 1193 |
|  |  | Renal failure not requiring dialysis | 1194 |
|  |  | Kidney nephropathy | 1519 |
|  |  | Immunoglobulin A (IgA) nephropathy | 1520 |
| 28 | Prostate disorders | Prostate problem (not cancer) | 1207 |
|  |  | Enlarged prostate | 1396 |
|  |  | Benign prostatic hypertrophy | 1516 |
| 29 | Glaucoma | Glaucoma | 1277 |
| 30 | Epilepsy | Epilepsy | 1264 |
| 31 | Dementia | Dementia/Alzheimer/cognitive impairment | 1263 |
| 32 | Psoriasis or eczema | Eczema/dermatitis | 1452 |
|  |  | Psoriasis | 1453 |
| 33 | Migraine | Migraine | 1265 |
| 34 | Chronic sinusitis | Chronic sinusitis | 1416 |
| 35 | Anorexia or bulimia | Anorexia, bulimia/other eating disorder | 1470 |
| 36 | Parkinson’s disease | Parkinson’s disease | 1262 |
| 37 | Multiple sclerosis | Multiple sclerosis | 1261 |
| 38 | Chronic fatigue syndrome | Chronic fatigue syndrome | 1482 |
| 39 | Endometriosis | Endometriosis | 1402 |
| 40 | Meniere disease | Meniere disease | 1421 |
| 41 | Pernicious anaemia | Pernicious anaemia | 1331 |
| 42 | Polycystic ovaries | Polycystic ovaries | 1350 |

*Self-report lifetime diagnosis by doctor recorded by interview (UK Biobank data field ID: 20002), except cancer diagnosis which was reported by touch-screen questionnaire. The list of disease groupings was based on Barnett et al (2012). In the present study, we did not include patients with diabetes at baseline.

**Table S3**. Gap statistic method information

| **gap** | **SE.sim** | **clusters** |  |
| --- | --- | --- | --- |
| 1.00492103307539 | 0.00307020727214925 | 1 |  |
| 0.954304781738079 | 0.02081526972778 | 2 |  |
| 0.922246674552937 | 0.00491895417882244 | 3 |  |
| 0.836364223373436 | 0.003414193338078 | 4 |  |
| 0.841539939639504 | 0.00643494575366929 | 5 |  |
| 0.836453099132834 | 0.00873430996395356 | 6 |  |
| 0.813937816589784 | 0.00610013075955172 | 7 |  |
| 0.805875127760426 | 0.00369670356464876 | 8 |  |
| 0.792791880873412 | 0.00547038000064489 | 9 |  |
| 0.7864821907653 | 0.00515774223904118 | 10 |  |

**Table S4**. Threshold effect analysis of the relationship of arterial stiffness index and diabetic microvascular complications

| **Arterial Stiffness Index (m/s)** | **Adjusted Model** | |
| --- | --- | --- |
|  | HR (95%CI) | *P* value |
| <9.92 | 0.945 (0.912,0.978) | 0.0014 |
| ≥9.92 | 1.015 (0.988,1.043) | 0.2694 |
| Likelihood ratio test |  | 0.002 |

HR, hazard ratio; CI, confidence interval; Adjusted for Model 3.

**Table S5**. Threshold effect analysis of the relationship of pulse pressure and diabetic microvascular complications

| **Pulse Pressure (10 mmHg)** | **Adjusted Model** | |
| --- | --- | --- |
|  | HR (95%CI) | *P* value |
| <7.12 | 1.036 (0.983,1.091) | 0.1846 |
| ≥7.12 | 1.161 (1.072,1.258) | < 0.001 |
| Likelihood ratio test |  | 0.011 |

HR, hazard ratio; CI, confidence interval; Adjusted for Model 3.

**Table S6.** Associations of arterial stiffness index and pulse pressure with diabetic microvascular complications in competing risk models

| **Variable** | **Model 1** | | **Model 2** | | **Model 3** | |
| --- | --- | --- | --- | --- | --- | --- |
|  | **SHR (95% CI)** | ***P* value** | **SHR (95% CI)** | ***P* value** | **SHR (95% CI)** | ***P* value** |
| **Arterial Stiffness Index** |  |  |  |  |  |  |
| Increase per 1 m/s | 0.98 (0.96, 0.99) | 0.001 | 0.97 (0.96, 0.99) | <0.001 | 0.98 (0.97, 0.99) | 0.022 |
| Tertile 1 | 1 (Ref) |  | 1 (Ref) |  | 1 (Ref) |  |
| Tertile 2 | 0.87 (0.79, 0.97) | 0.009 | 0.85 (0.77, 0.94) | 0.002 | 0.87 (0.78, 0.98) | 0.017 |
| Tertile 3 | 0.82 (0.74, 0.91) | <0.001 | 0.81 (0.73, 0.9) | <0.001 | 0.88 (0.79, 0.98) | 0.015 |
| *P* for trend |  | <0.001 |  | <0.001 |  | 0.017 |
| **Pulse Pressure** |  |  |  |  |  |  |
| Increase per 10 mmHg | 1.05 (1.02, 1.08) | <0.001 | 1.06 (1.03, 1.09) | <0.001 | 1.07 (1.04, 1.10) | <0.001 |
| Tertile 1 | 1 (Ref) |  | 1 (Ref) |  | 1 (Ref) |  |
| Tertile 2 | 1 (0.9, 1.12) | 0.943 | 1.02 (0.92, 1.14) | 0.673 | 1.08 (0.97, 1.21) | 0.152 |
| Tertile 3 | 1.14 (1.03, 1.27) | 0.016 | 1.17 (1.05, 1.3) | 0.004 | 1.24 (1.11, 1.38) | <0.001 |
| *P* for trend |  | 0.011 |  | 0.003 |  | <0.001 |

Model 1 was adjusted for age, sex, and ethnic groups; Model 2 was adjusted for drinking status, smoking status, educational level, Townsend Deprivation Index, and body mass index; Model 3 was further adjusted for albumin, urea, cholesterol, creatinine, C-reactive protein, glycated hemoglobin, and number of long-term conditions. HR, hazard ratio; CI, confidence interval; Ref, reference. Subdistribution hazard ratios were estimated using Fine–Gray models accounting for the competing risk of death.

**Table S7**. Threshold effect analysis of the relationship of arterial stiffness index and diabetic kidney disease

| **Arterial Stiffness Index (m/s)** | **Adjusted Model** | |
| --- | --- | --- |
|  | HR (95%CI) | *P* value |
| <11.53 | 0.934 (0.904,0.966) | < 0.001 |
| ≥11.53 | 1.038 (0.986,1.093) | 0.1569 |
| Likelihood ratio test |  | 0.001 |

HR, hazard ratio; CI, confidence interval; Adjusted for Model 3.

**Table S8**. Threshold effect analysis of the relationship of pulse pressure and diabetic kidney disease

| **Pulse Pressure (10 mmHg)** | **Adjusted Model** | |
| --- | --- | --- |
|  | HR (95%CI) | *P* value |
| <5.7 | 0.879 (0.764,1.012) | 0.0724 |
| ≥5.7 | 1.151 (1.089,1.217) | < 0.001 |
| Likelihood ratio test |  | 0.001 |

HR, hazard ratio; CI, confidence interval; Adjusted for Model 3.

**Table S9.** Associations of hemodynamic phenotypes and diabetic microvascular complications in the complete-case analysis

| **Hemodynamic phenotypes** | **Adjusted Hazard Ratio (95% CI)** | | |
| --- | --- | --- | --- |
|  | **Model 1** | **Model 2** | **Model 3** |
| Low ASI-PP | 1(Ref) | 1(Ref) | 1(Ref) |
| High ASI | 0.9 (0.8, 1.02) | 0.91 (0.8, 1.04) | 0.96 (0.85, 1.09) |
| High PP | 1.13 (1.01, 1.27) | 1.16 (1.03, 1.3) | 1.15 (1.02, 1.29) |

Model 1 was adjusted for age, sex, and ethnic groups; Model 2 was adjusted for drinking status, smoking status, educational level, Townsend Deprivation Index, and body mass index; Model 3 was further adjusted for albumin, urea, cholesterol, creatinine, C-reactive protein, glycated hemoglobin, and number of long-term conditions. CI, confidence interval; Ref, reference; ASI, arterial stiffness index; PP, pulse pressure. Hazard ratios were estimated using multivariable Cox proportional hazards models.

**Table S10.** Associations of arterial stiffness index and pulse pressure with diabetic microvascular complications in the complete-case analysis

| **Variable (events/total)** | **Model 1** | | **Model 2** | | **Model 3** | |
| --- | --- | --- | --- | --- | --- | --- |
|  | **HR (95% CI)** | ***P* value** | **HR (95% CI)** | ***P* value** | **HR (95% CI)** | ***P* value** |
| **Arterial Stiffness Index** |  |  |  |  |  |  |
| Increase per 1 m/s | 0.98 (0.97, 1) | 0.029 | 0.98 (0.96, 1) | 0.013 | 0.99 (0.97, 1) | 0.153 |
| Tertile 1 (818/3052) | 1 (Ref) |  | 1 (Ref) |  | 1 (Ref) |  |
| Tertile 2 (759/3053) | 0.83 (0.73, 0.93) | 0.002 | 0.8 (0.71, 0.9) | <0.001 | 0.82 (0.73, 0.93) | 0.002 |
| Tertile 3 (772/3058) | 0.86 (0.76, 0.97) | 0.011 | 0.84 (0.75, 0.95) | 0.004 | 0.91 (0.8, 1.02) | 0.113 |
| *P* for trend |  | 0.012 |  | 0.005 |  | 0.125 |
| **Pulse Pressure** |  |  |  |  |  |  |
| Increase per 10 mmHg | 1.08 (1.05, 1.12) | <0.001 | 1.09 (1.06, 1.12) | <0.001 | 1.1 (1.06, 1.13) | <0.001 |
| Tertile 1 (642/2967) | 1 (Ref) |  | 1 (Ref) |  | 1 (Ref) |  |
| Tertile 2 (743/3039) | 1.04 (0.92, 1.18) | 0.518 | 1.07 (0.94, 1.22) | 0.3 | 1.09 (0.96, 1.24) | 0.17 |
| Tertile 3 (964/3157) | 1.23 (1.09, 1.39) | 0.001 | 1.27 (1.12, 1.44) | <0.001 | 1.3 (1.15, 1.47) | <0.001 |
| *P* for trend |  | 0.001 |  | <0.001 |  | <0.001 |

Model 1 was adjusted for age, sex, and ethnic groups; Model 2 was adjusted for drinking status, smoking status, educational level, Townsend Deprivation Index, and body mass index; Model 3 was further adjusted for albumin, urea, cholesterol, creatinine, C-reactive protein, glycated hemoglobin, and number of long-term conditions. HR, hazard ratio; CI, confidence interval; Ref, reference. Hazard ratios were estimated using multivariable Cox proportional hazards models.

**Table S11.** Associations of hemodynamic phenotypes and diabetic microvascular complications excluding events occurring within the first 2 years of follow-up

| **Hemodynamic phenotypes** | **Adjusted Hazard Ratio (95% CI)** | | |
| --- | --- | --- | --- |
|  | **Model 1** | **Model 2** | **Model 3** |
| Low ASI-PP | 1(Ref) | 1(Ref) | 1(Ref) |
| High ASI | 0.89 (0.8, 0.99) | 0.9 (0.8, 1) | 0.96 (0.86, 1.07) |
| High PP | 1.11 (1.01, 1.23) | 1.14 (1.03, 1.26) | 1.15 (1.04, 1.27) |

Model 1 was adjusted for age, sex, and ethnic groups; Model 2 was adjusted for drinking status, smoking status, educational level, Townsend Deprivation Index, and body mass index; Model 3 was further adjusted for albumin, urea, cholesterol, creatinine, C-reactive protein, glycated hemoglobin, and number of long-term conditions. CI, confidence interval; Ref, reference; ASI, arterial stiffness index; PP, pulse pressure. Hazard ratios were estimated using multivariable Cox proportional hazards models.

**Table S12.** Associations of arterial stiffness index and pulse pressure with diabetic microvascular complications excluding events occurring within the first 2 years of follow-up

| **Variables** | **Model 1** | | **Model 2** | | **Model 3** | |
| --- | --- | --- | --- | --- | --- | --- |
|  | **HR (95% CI)** | ***P* value** | **HR (95% CI)** | ***P* value** | **HR (95% CI)** | ***P* value** |
| **Arterial Stiffness Index** |  |  |  |  |  |  |
| Increase per 1 m/s | 0.98 (0.96, 0.99) | 0.002 | 0.97 (0.96, 0.99) | <0.001 | 0.98 (0.97, 1) | 0.014 |
| Tertile 1 | 1 (Ref) |  | 1 (Ref) |  | 1 (Ref) |  |
| Tertile 2 | 0.86 (0.78, 0.95) | 0.004 | 0.83 (0.75, 0.92) | <0.001 | 0.87 (0.78, 0.96) | 0.008 |
| Tertile 3 | 0.83 (0.75, 0.92) | <0.001 | 0.81 (0.73, 0.9) | <0.001 | 0.88 (0.79, 0.98) | 0.016 |
| *P* for trend |  | <0.001 |  | <0.001 |  | 0.017 |
| **Pulse Pressure** |  |  |  |  |  |  |
| Increase per 10 mmHg | 1.05 (1.02, 1.08) | <0.001 | 1.06 (1.03, 1.09) | <0.001 | 1.07 (1.04, 1.1) | <0.001 |
| Tertile 1 | 1 (Ref) |  | 1 (Ref) |  | 1 (Ref) |  |
| Tertile 2 | 0.97 (0.87, 1.08) | 0.554 | 0.98 (0.88, 1.1) | 0.745 | 1.03 (0.92, 1.15) | 0.606 |
| Tertile 3 | 1.12 (1, 1.25) | 0.045 | 1.14 (1.02, 1.27) | 0.02 | 1.21 (1.08, 1.34) | 0.001 |
| *P* for trend |  | 0.028 |  | 0.012 |  | 0.001 |

Model 1 was adjusted for age, sex, and ethnic groups; Model 2 was adjusted for drinking status, smoking status, educational level, Townsend Deprivation Index, and body mass index; Model 3 was further adjusted for albumin, urea, cholesterol, creatinine, C-reactive protein, glycated hemoglobin, and number of long-term conditions. HR, hazard ratio; CI, confidence interval; Ref, reference. Hazard ratios were estimated using multivariable Cox proportional hazards models.

**Table S13.** Stratified analyses

| **Variable** | **HR (95%CI)** | ***P* value** | ***P* for interaction** |
| --- | --- | --- | --- |
| Female |  |  | 0.539 |
| Low ASI-PP | 1(Ref) |  |  |
| High ASI | 1.21 (1, 1.45) | 0.049 |  |
| High PP | 1.36 (1.16, 1.59) | <0.001 |  |
| Male |  |  |  |
| Low ASI-PP | 1(Ref) |  |  |
| High ASI | 1.07 (0.93, 1.22) | 0.349 |  |
| High PP | 1.39 (1.22, 1.57) | <0.001 |  |
| Age <65 years |  |  | 0.142 |
| Low ASI-PP | 1(Ref) |  |  |
| High ASI | 1.19 (0.97, 1.47) | 0.101 |  |
| High PP | 1.32 (1.07, 1.62) | 0.008 |  |
| Age ≥65 years |  |  |  |
| Low ASI-PP | 1(Ref) |  |  |
| High ASI | 0.93 (0.82, 1.05) | 0.247 |  |
| High PP | 1.18 (1.05, 1.33) | 0.004 |  |
| HbA1c <48 mmol/mol |  |  | 0.753 |
| Low ASI-PP | 1(Ref) |  |  |
| High ASI | 1.07 (0.88, 1.3) | 0.512 |  |
| High PP | 1.43 (1.2, 1.7) | <0.001 |  |
| HbA1c ≥48 mmol/mol |  |  |  |
| Low ASI-PP | 1(Ref) |  |  |
| High ASI | 1.09 (0.96, 1.24) | 0.2 |  |
| High PP | 1.36 (1.21, 1.53) | <0.001 |  |
| Non hypertension |  |  | 0.275 |
| Low ASI-PP | 1(Ref) |  |  |
| High ASI | 1.12 (0.93, 1.36) | 0.237 |  |
| High PP | 1.52 (1.26, 1.84) | <0.001 |  |
| Hypertension |  |  |  |
| Low ASI-PP | 1(Ref) |  |  |
| High ASI | 1.05 (0.92, 1.2) | 0.456 |  |
| High PP | 1.27 (1.13, 1.42) | <0.001 |  |
| Non coronary heart disease |  |  | 0.461 |
| Low ASI-PP | 1(Ref) |  |  |
| High ASI | 1.11 (0.98, 1.25) | 0.099 |  |
| High PP | 1.37 (1.23, 1.54) | <0.001 |  |
| Coronary heart disease |  |  |  |
| Low ASI-PP | 1(Ref) |  |  |
| High ASI | 0.98 (0.78, 1.24) | 0.866 |  |
| High PP | 1.37 (1.11, 1.69) | 0.003 |  |

Adjusted for Model 3. Ref, reference; HbA1c, glycated hemoglobin.

**Table S14.** Baseline characteristics of participants included in the analysis and those excluded because of unavailable ASI or PP measurements

| **Participant characteristics** | **Total** | **Included** | **Excluded** | ***P* value** |
| --- | --- | --- | --- | --- |
|  | **n=26778** | **n=9163** | **n=17615** |  |
| **Age, y** | 59.7 ± 7.1 | 59.7 ± 7.1 | 59.7 ± 7.1 | 0.464 |
| **Gender, n (%)** |  |  |  | 0.831 |
| Female | 10199 (38.1) | 3498 (38.2) | 6701 (38) |  |
| Male | 16579 (61.9) | 5665 (61.8) | 10914 (62) |  |
| **Ethnic groups, n (%)** |  |  |  | < 0.001 |
| White | 23144 (87.2) | 7155 (79.1) | 15989 (91.4) |  |
| Mix | 175 ( 0.7) | 81 (0.9) | 94 (0.5) |  |
| Asian or Asian British | 1725 ( 6.5) | 1005 (11.1) | 720 (4.1) |  |
| Black or Black British | 946 ( 3.6) | 525 (5.8) | 421 (2.4) |  |
| Chinese or Others | 558 ( 2.1) | 282 (3.1) | 276 (1.6) |  |
| **Drinking status, n (%)** |  |  |  | < 0.001 |
| ≥ 3 times per week | 7987 (30.0) | 2666 (29.3) | 5321 (30.3) |  |
| < 3 times per week | 9495 (35.6) | 3117 (34.2) | 6378 (36.4) |  |
| Not current | 9170 (34.4) | 3325 (36.5) | 5845 (33.3) |  |
| **Smoking status, n (%)** |  |  |  | < 0.001 |
| Non-smoker | 12029 (45.4) | 4356 (48.2) | 7673 (44) |  |
| Former smoker | 11396 (43.0) | 3712 (41) | 7684 (44) |  |
| Current smoker | 3070 (11.6) | 975 (10.8) | 2095 (12) |  |
| **Educational level, n (%)** |  |  |  | < 0.001 |
| College or University degree | 6139 (23.6) | 2329 (26.2) | 3810 (22.3) |  |
| Any other qualification | 12586 (48.5) | 4351 (48.9) | 8235 (48.2) |  |
| No qualification | 7248 (27.9) | 2217 (24.9) | 5031 (29.5) |  |
| **Townsend deprivation index** | -1.2 (-3.2, 2.1) | -0.8 (-2.9, 2.2) | -1.4 (-3.3, 2.1) | < 0.001 |
| **BMI, (kg/m^2^)** | 31.5 ± 5.8 | 31.3 ± 5.7 | 31.7 ± 5.9 | < 0.001 |
| **Albumin, (g/L)** | 44.9 ± 2.9 | 45.1 ± 2.9 | 44.8 ± 2.8 | < 0.001 |
| **Urea, (mg/dL)** | 5.8 ± 1.9 | 5.7 ± 1.8 | 5.9 ± 1.9 | < 0.001 |
| **Cholesterol, (mg/dL)** | 4.6 ± 1.1 | 4.6 ± 1.1 | 4.6 ± 1.1 | 0.393 |
| **Creatinine, (umol/L)** | 74.9 ± 24.8 | 74.9 ± 25.2 | 74.9 ± 24.6 | 0.897 |
| **C-reactive protein, (mg/dL)** | 2.0 (0.9, 4.2) | 1.9 (0.9, 4.0) | 2.0 (1.0, 4.2) | < 0.001 |
| **HbA1c, (mmol/mol)** | 53.8 ± 14.7 | 53.2 ± 14.4 | 54.1 ± 14.9 | < 0.001 |
| **No. of long-term conditions** | 2.7 ± 1.4 | 2.6 ± 1.4 | 2.7 ± 1.4 | < 0.001 |

Continuous variables are presented as mean ± standard deviation and median (interquartile range), and categorical variables as counts (percentages). BMI, body mass index; HbA1c, glycated hemoglobin; ASI, arterial stiffness index; PP, pulse pressure. The data after using multiple imputation.
